# Supplementary material for: Bright and durable scintillation from colloidal quantum shells
Source: Nat Commun. 2024 May 20;15:4274. doi: 10.1038/s41467-024-48351-9 (PMC11106345; doi:10.1038/s41467-024-48351-9)
Supplement: Supplementary file 1 — Supplementary Information [file 41467_2024_48351_MOESM1_ESM.pdf]

Supplementary Information

Bright and Durable Scintillation from Colloidal Quantum Shells

Burak Guzelturk[1*], Benjamin T. Diroll[2*], James P. Cassidy[3], Dulanjan Harankahage[3], Muchuan Hua,[2] Xiao-Min Lin[2], Vasudevan Iyer[4], Richard D. Schaller[2,5], Benjamin J. Lawrie[4,6], and Mikhail Zamkov[3*]

Structural properties of the colloidal quantum shells:
Supplementary Figure 1 shows transmission electron micrographs
Supplementary Figure 2 shows X-ray diffraction

Spectral properties:
Supplementary Figure 3 shows radioluminescence vs. photoluminescence spectra
Supplementary Figure 4 shows spectral reflectance for film thickness measurement
Supplementary Figure 5 shows radioluminescence spectra (raw data)

Supplementary Note 1: Pulse height spectrum measurements
Supplementary Figure 6 shows pulse height spectra measurements

Radioluminescence properties:
Supplementary Figure 7 shows radioluminescence rise and decays
Supplementary Figure 8 shows X-ray flux dependent radioluminescence

Investigation of radiation effects:
Supplementary Figure 9 shows photoluminescence before and after X-ray irradiation
Supplementary Figure 10 shows scanning electron micrograph before and after X-ray irradiation

Supplementary Table 1 shows film thicknesses
Supplementary Table 2 shows light yield and decay lifetime of various scintillator materials

Supplementary Table 3 shows radioluminescence decay components

Optical properties:

Supplementary Figure 11 shows scintillation imaging with NaI:Tl

Supplementary Figure 12 shows photoluminescence decay dynamics

Supplementary Figure 13 shows low vs. high fluence photoluminescence spectra

Supplementary Figure 14 shows fluence dependent integrated photoluminescence intensity

Supplementary Note 2: Variable power scaling model for exciton relaxation

Supplementary Table 4 shows parameters used in the model

Supplementary Table 5 shows Biexciton Auger-Meitner lifetimes

Supplementary Figure 15 shows temporal evolution of exciton population

Supplementary Figure 16 shows experimental measurement of exciton density vs. time

Supplementary Note 3: Estimation of secondary electron deposition spread

Supplementary Figure 17 shows a sketch of primary excitation and secondary electron spread

Supplementary Note 4: Cathodoluminescence properties

Supplementary Figure 18 shows CL from QSs in comparison to gold reference with lifetimes

Supplementary Figure 19 shows CL vs PL from QSs

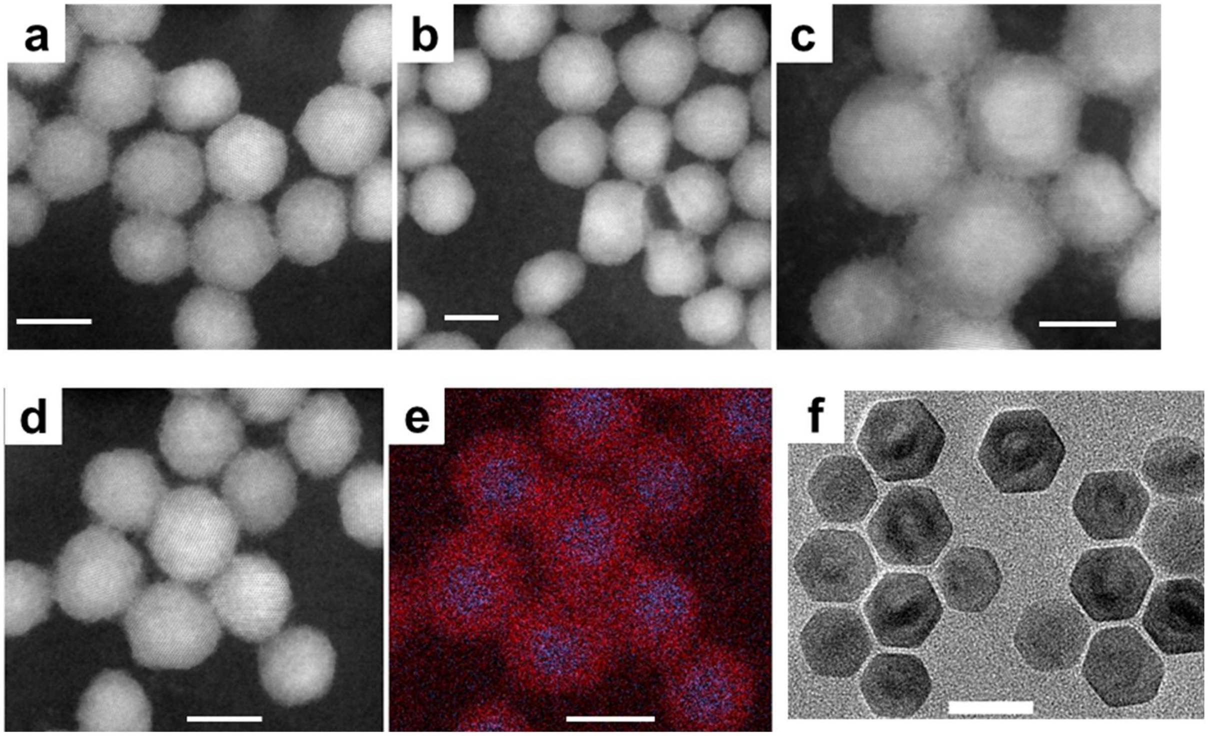

Supplementary Figure 1: **Electron microscopy images.** (a-d) Dark field TEM images of quantum shells (QSs). Core sizes are (a) 4.5 nm (b) 6.0 nm, (c) 8.2 nm, and (d) 4.5 nm. (e) is an overlayed EDX map of the sulfur (red) and selenium (blue) detected in the same spot as (d). The scale bar for (a-e) is 10 nm. (f) High resolution TEM image of the QSs showing hexagonal shapes due to wurtzite structure. The scale bar is 25 nm.

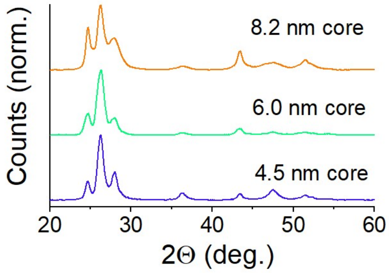

Supplementary Figure 2: **X-ray diffraction measurements.** X-ray diffraction patterns of three QSs used in this work.

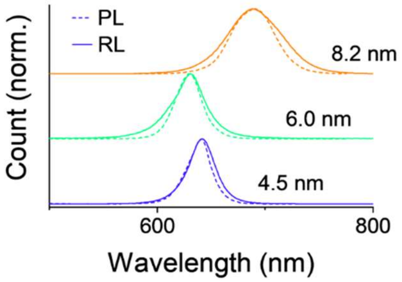

Supplementary Figure 3: **Radioluminescence vs. Photoluminescence spectra.** Comparison of photoluminescence and radioluminescence of the QS samples. Difference in broadening may be due to use of different spectrometers for measurements with varying input slit width.

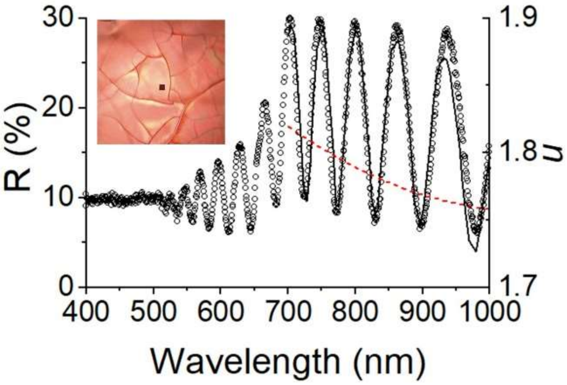

Supplementary Figure 4: **Spectral reflectance spectrum.** Reflectance spectrum of 8.2 nm core QS sample (left axis) with interference fringes apparent in the transparent region. The ellipsometric index of the sample used to fit the interference fringes is shown in the red dashed line (scale at right). The inset camera image shows the film, which has cracks from drying. The black square is the measurement point.

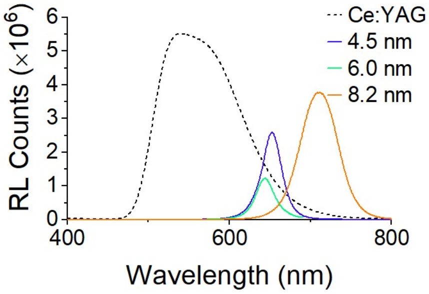

Supplementary Figure 5: **Radioluminescence spectra.** Raw radioluminescence of QS samples and Ce:YAG scintillation standard measured at normal incidence. Data in Figure 2 are adjusted for measured sample thickness.

Supplementary Note 1: Pulse height spectrum measurements of the light yield

We carry out an alternative measurement to characterize the light yield (LY) of the quantum shells. For this, we performed pulse height spectrum measurements of our QS scintillator film. To measure the pulse height spectrum and determine an absolute LY, we followed the methodology by Moszynski et al.[1].

Briefly, we used a silicon avalanche photodiode (si-APD) with a 1×1 cm active area and a ^{55}Fe radiation source emitting monochromatic X-rays at ~5.9 keV. The si-APD was covered with a beryllium cap (thickness 200 µm) to block any ambient light while transmitting the 5.9 keV radiation. Si-APD output was fed into a high-speed GHz pre-amplifier (HAS-X-2-40, Femto) and then into an amplifier (DH-PCA-100, Femto). The amplified output is then fed into a high bandwidth oscilloscope (MSO9404A, Keysight) for analyzing the individual pulse heights. Supplementary Figure 6 shows the pulse height spectra measured for two different cases while keeping the detector amplification and other settings the same:

First, only ^{55}Fe radiation source coupled to si-APD (Supplementary Figure 6a)

Second, QS scintillator placed in front of the si-APD between the silicon and beryllium window (Supplementary Figure 6b). QS film (5 µm) was on placed on 300 µm thick glass. Direct transmission of 5.9 keV X-rays from ^{55}Fe is mostly blocked by the glass substate as transmission coefficient is 2×10^{-3}, or the attenuation length is 50 µm. Glass itself has substantially weaker scintillation, and the bare glass radioluminescence is below the noise level.

We use the first measurement to calibrate the pulse height versus number of electron-hole pairs generated in the silicon at 5.9 keV energy. Generated electron – hole pairs ($n_{e-h\_pair\_55Fe}$) is equal to

$$n_{\text{e-h\_pair\_55Fe}} = \frac{E_{\text{x-ray}}}{E_{\text{impact\_ionization}}} = \frac{5.8988\ \text{keV}}{3.65\ \text{eV}} = 1616 \qquad (1),$$

where $E_{impact\_ionization}$ is 3.65 eV that is the multi-carrier generation threshold of silicon[2]. Then, we use the second measurement to estimate the number of electron-hole pairs created by the visible photons of the QS scintillator ($n_{e-h\_pair\_QS}$). We compare the pulse height (PH) ratio to estimate $n_{\text{e-h\_pair\_QS}} = \frac{PH_{\text{QS}}}{PH_{\text{55Fe}}} \times n_{\text{e-h\_pair\_55Fe}} = \frac{1}{5} \times 1616 = 323$ ($\pm 4\%$) e-h pairs. The LY of the QS scintillator is then simply calculated by

$$LY = n_{\text{e-h\_pair\_QS}} \times \frac{1}{QE_{\text{si\_APD}}} \times \frac{1}{\eta_{\text{light\_coupling}}} \times \frac{1000}{E_{x-ray}} \qquad (2),$$

where $QE_{\text{si\_APD}}$ is the quantum efficiency of the APD at the emission wavelength of the QS (~700 nm), $\eta_{\text{light\_coupling}}$ is the efficiency of the coupling of the scintillated visible light into the APD active area. $QE_{\text{si\_APD}}$ is 85% at 700 nm. $\eta_{\text{light\_coupling}}$ is ~80% ($\pm 10\%$) due to light trapping by glass substate's waveguiding modes[3]. With the pulse height spectrum method, we

determine the LY to be ~80,000 $\pm$ 8,600 (mean $\pm$ standard deviation) photons MeV^{-1}. This is in agreement, within 15%, with our comparative LY measurements that yielded 70,000 $\pm$ 13,300 photons MeV^{-1}.

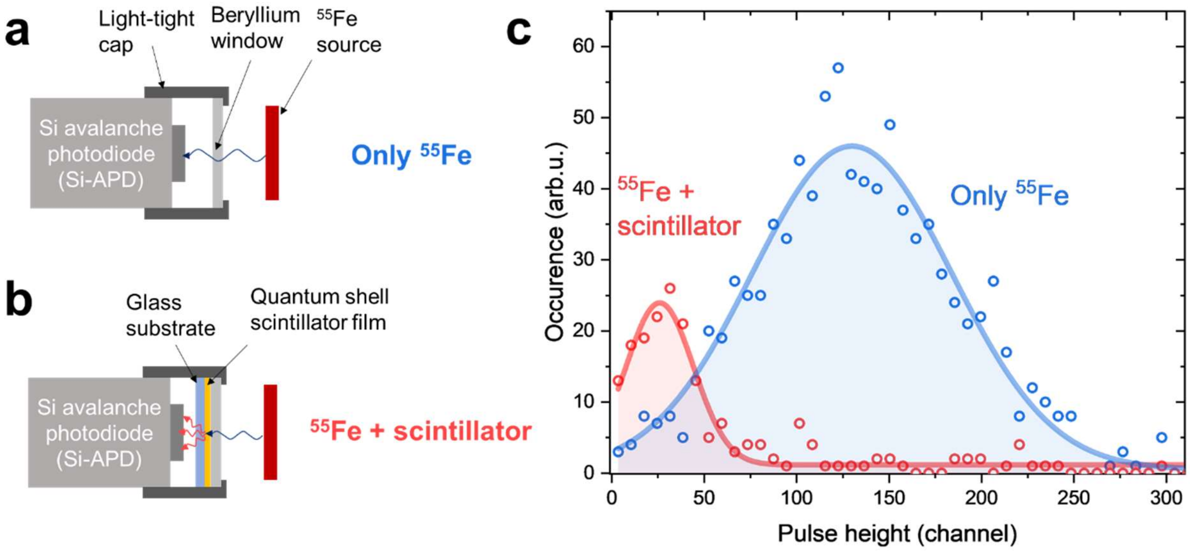

Supplementary Figure 6: **Pulse height spectrum measurements.** Two different configurations used for pulse height measurements using a silicon APD as the detector: (a) only ^{55}Fe radiation source, (b) quantum shell (QS) scintillator film placed in front of the si-APD with ^{55}Fe radiation source. (c) Pulse height spectra of both cases, circles are experimental data and solid curves are the Gaussian fits. Measurement without scintillator (only ^{55}Fe source) lasted 30 seconds, while it took 7 minutes with the scintillator inserted due to difference in occurrence statistics in two difference cases. Y-axis is not normalized. Only ^{55}Fe measurement (blue) is used to calibrate the pulse height vs. number of electron-hole pairs. Measurement with QS film (red) is used to determine the light yield of the QS scintillator. Gain of the si-APD is the same for both measurements.

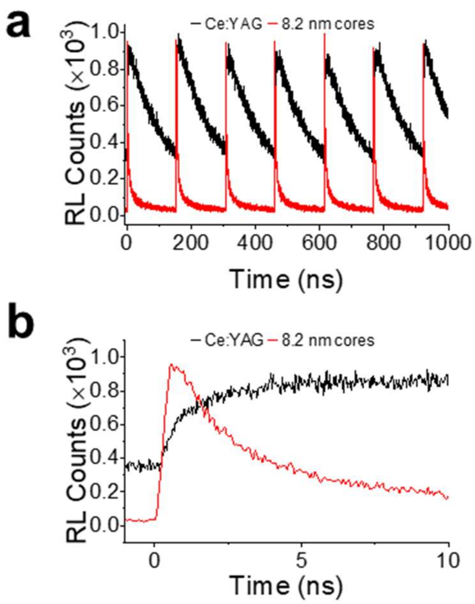

Supplementary Figure 7: **Radioluminescence temporal dynamics.** (a) Radioluminescence of Ce:YAG and 8.2 nm core QSs as a function of time. (b) Early time window of radioluminescence dynamics showing rise of the signals.

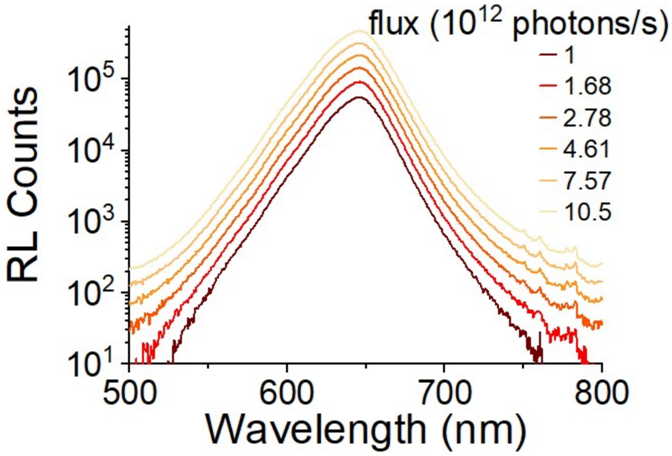

Supplementary Figure 8: **X-ray flux dependence of radioluminesce.** Spectra of 4.5 nm core QSs with several x-ray photon flux intensities.

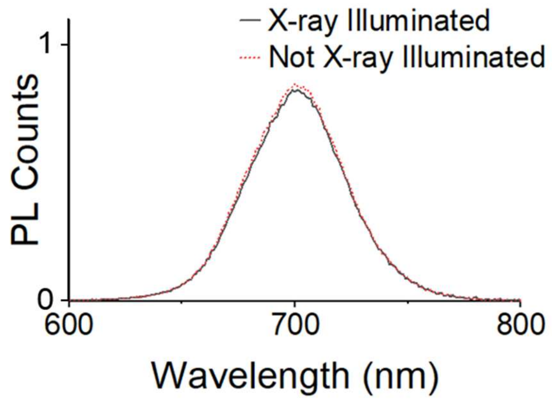

Supplementary Figure 9: **Photoluminescence stability.** Emission spectra of two different regions of the sample: high-flux X-ray excitation region vs. no X-ray excited region.

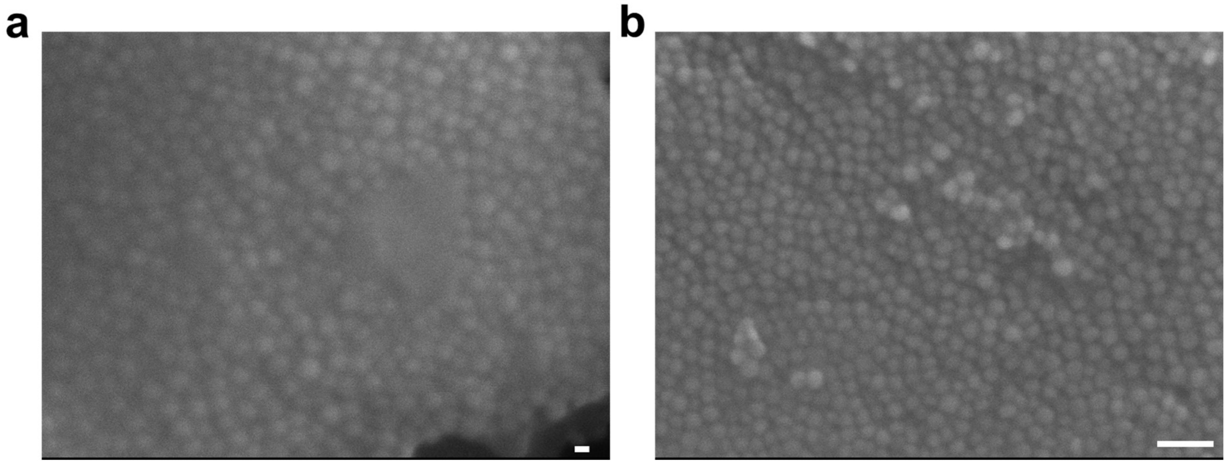

Supplementary Figure 10: **Structural stability.** Scanning electron microscopy images of the (a) high-flux X-ray irritated area, (b) un-exposed region. Scale bar is 10 nm in (a), and 50 nm in (b). SEM images and PL measurements before and after high-flux X-ray irradiation show that the micro and nanostructure of the QSs is preserved.

Supplementary Table 1. The QS film thicknesses

sample	thickness (nm)	error (nm)
core: 4.5 nm	3429	444
core: 6.0 nm	3892	288
core: 8.2 nm	1387	127
Ce:YAG	100000	500

Supplementary Table 2. Tabulated Scintillator Properties

Sample	λ_{em} (nm)	Light Yield (ph keV^{-1})	τ (ns)	Ref.	Class
(EDBE)PbCl4	520	120	7.9	10.1038/srep37254	cryo
(3-FC6H5C2H4NH3)2PbBr4 single crystal	430	12	6.9	https://doi.org/10.1016/j.optmat.2020.109686	inorganic
(C38H34P2)MnBr4 single crystal	517	80	318000	10.1038/s41467-020-18119-y	inorganic
(C6H5C2H4NH3)2Pb0.75Ba0.25Br4 single crystal	440	19	9.2	10.7567/1347-4065/ab515d	inorganic
(C6H5C2H4NH3)2Pb0.975Mn0.025Br4 single crystal	410	1.6	0.08	10.7567/1347-4065/ab2e7c	inorganic
(C6H5C2H4NH3)2Pb0.9Sr0.1Br4 single crystal	440	19.7	4.54	https://doi.org/10.1016/j.nima.2018.10.050	inorganic
Li-doped (PEA)2PbBr4 single crystal	436	11	11	10.1038/s43246-020-0038-x	inorganic
PhE-PbBr4	475	8.36	9.9	10.1063/1.3059562	inorganic
(PPN)2SbCl5 single crystal	635	49	4100	https://doi.org/10.1016/j.matt.2020.05.018	inorganic
BaF2	310	2	0.6	https://doi.org/10.1016/0167-5087(83)91254-1	inorganic
BaMgF4	210	1.3	0.57	10.1016/j.nima.2010.06.218	inorganic
bismuth germanate (BGO)	450	0.55	60	https://doi.org/10.1016/0029-554X(81)90521-8	inorganic
CdWO4	495	20	2000	J.Phys.Stud. 14 (2010) 4, 4201. SCINTILLATORS FOR CRYOGENIC APPLICATIONS: STATE-OF-ART	inorganic
Cs2NaEuCl6 powder crysta	600	1.25	160000	10.1007/s11426-018-9308-2	inorganic

Cs2NaTbCl6 powder crystal	548	46.6	760000	10.1007/s11426-018-9308-2	inorganic
Cs2ZnCl4		0.63	1.8	10.7567/APEX.7.062602	inorganic
Cs3Cu2I5 single crysta	440	32	51	10.1002/pssr.202000374	inorganic
Cs3Cu2I5:Tl single crystal	500	51	304	10.1021/acsami.0c09047	inorganic
Cs4CaI6:Eu single crystal	474	51.8	2100	https://doi.org/10.1016/j.jcrysgro.2018.01.017	inorganic
Cs4EuBr6 single crystal	462	78	3720	10.1039/c8tc01458b	inorganic
Cs4EuI6 single crystal	470	53	1290	10.1039/c8tc01458b	inorganic
Cs4SrI6:Eu single crysta	474	62.3	1900	https://doi.org/10.1016/j.jcrysgro.2018.01.017	inorganic
CsF	390	0.52	2	https://doi.org/10.1016/0167-5087(83)90194-1	inorganic
CsI	315	0.967		https://doi.org/10.1016/j.nima.2020.164801; 10.1088/1361-6560/ab63b4	inorganic
CsI:Tl	550	60	1000	https://doi.org/10.1016/0168-9002(93)91015-F	inorganic
CsPbCl3 single crystal	415	0.33	0.3	https://doi.org/10.1016/j.nima.2008.04.079	inorganic
CsPbCl3 single crystal	440	1.2	0.39	10.7567/JJAP.55.02BC20	inorganic
GOS:Tb	545	60	600000	https://doi.org/10.1016/j.nima.2015.08.041	inorganic
K2CuBr3 single crystal	391	23.806	64.3	10.1021/acsaelm.0c00414	inorganic
LaBr3:Ce	358	61	35	https://doi.org/10.1016/S0168-9002(02)00712-X; https://doi.org/10.1016/j.jlumin.2021.118534	inorganic
LSO:Ce	420	40	40	10.1109/23.159655	inorganic
MaPbBr0.05Cl2.95	420	18	0.14	10.1021/acsami.9b10367	inorganic
NaI:Tl	415	43	230	, https://doi.org/10.1016/j.nima.2014.02.045	inorganic
PbWO4	475	0.14	10	https://doi.org/10.1016/0168-9002(95)00589-7	inorganic
Rb2CuBr3 single crystal	385	91.056	41400	10.1002/adma.201904711	inorganic
Rb2CuCl3 single crystal	401	16.6	11.3	10.1021/acs.jpclett.0c00161	inorganic
YAP: Ce	340	17.2	27	10.1016/0168-583X(91)95605	inorganic
BaF2	220	1.8	0.6	St. Gobain	inorganic
BaF2	310	10	630	St. Gobain	inorganic
BGO	480	10	300	St. Gobain	inorganic
CaF2:Eu	435	19	940	St. Gobain	inorganic
CdWO4	475	15	14000	St. Gobain	inorganic
CLLB	420	43	180	St. Gobain	inorganic
CsI	315	2	16	St. Gobain	inorganic

CsI:Na	420	41	630	St. Gobain	inorganic
CsI:Tl	550	54	1000	St. Gobain	inorganic
LaBr3(ce)	380	63	16	St. Gobain	inorganic
LaBr3(Ce+Sr)	385	73	25	St. Gobain	inorganic
LYSO	420	33	36	St. Gobain	inorganic
NaI:Tl	415	38	250	St. Gobain	inorganic
NaIL	419	35	240	St. Gobain	inorganic
YAG:Ce	550	8	70	St. Gobain	inorganic
ZnS(ag)	310	50	110	St. Gobain	inorganic
CsPbBr3	525	50	1	10.1038/s41598-020-65672-z	cryo
MAPbBr3	575	152	0.8	10.1038/srep37254	cryo
MAPbBr3	575	116	1.5	10.1039/C9MH00281B	cryo
MAPbI3	780	270	4.3	10.1038/srep37254	cryo
CdSe/CdS NPLs	530	0.275	0.077	10.1016/j.jlumin.2019.116613, 10.1088/1748-0221/11/10/P10015	nano
CdTe NPLs	510	0.5			nano
Cs3Cu2I5 nanocrystals	445	79.279	1920	10.1002/advs.202000195	nano
CsPbBr3 nanosheets	520	21	2	10.1021/acsnano.8b09484	nano
CsPbBr3@Cs4PbBr6	520	6	3	10.1021/acsnano.9b06114	nano
CsPbBr3@Cs4PbBr6	520	64	1.4	10.1039/D0NR00772B	nano
CsPbBr3+fluoride treatment	535	9	2	10.1038/s41566-022-01103-x	nano
gQDs CdSe/CdS	650	23	0.895	10.1088/1748-0221/11/10/P10015	nano
ZnO:Ga	390	0.5		https://doi.org/10.1016/j.jlumin.2019.116613	nano
BC-400	423	12.7075	2.4	St. Gobain	plastic
BC-404	408	13.294	1.8	St. Gobain	plastic
BC-408	425	12.512	2.1	St. Gobain	plastic
BC-412	434	11.73	3.3	St. Gobain	plastic
BC-416	434	7.429	4	St. Gobain	plastic
BC-418	391	13.1	1.4	St. Gobain	plastic
BC-420	391	12.512	1.5	St. Gobain	plastic
BC-422	370	10.7525	1.6	St. Gobain	plastic
BC-422q	370	2.1505	0.7	St. Gobain	plastic
BC-428	480	7.038	12.5	St. Gobain	plastic
BC-430	580	8.7975	16.8	St. Gobain	plastic
BC-440	434	11.73	3.3	St. Gobain	plastic
BC-440M	428	11.73	3.3	St. Gobain	plastic
BC-444	428	8.0155	285	St. Gobain	plastic
BC-452	424	9.384	2.1	St. Gobain	plastic
BC-490	425	10.7525	2.3	St. Gobain	plastic
BC-498	423	12.7075	2.4	St. Gobain	plastic
YAG:Ce	549	30	70	https://www.crytur.cz/materials/yagce/	inorganic
BC-418	391	9.1	1.2	https://doi.org/10.1016/j.nima.2020.164801	plastic
BC-422	370	7.5	1.3	https://doi.org/10.1016/j.nima.2020.164801	plastic

Bi- and lithium loaded plastic	385	5	2	https://doi.org/10.1016/j.nima.2015.01.008	plastic
EJ-232 (Eljen)	370	8.4	1.6	10.1109/TNS.2020.3010469	plastic
EJ-232q (Eljen)	370	2.9	0.7	10.1109/TNS.2020.3010469	plastic
Hf-doped polymer (st gobain Bc452)	420	5.16	2.5	10.1063/1.4875025	plastic
NE142 (St. gobain BC-452)	370	4.3	2.1	10.1063/1.4875025	plastic
EJ-200	425	10	2.1	Eljen	plastic
EJ-204	408	10.4	1.8	Eljen	plastic
EJ-208	435	9.2	3.3	Eljen	plastic
EJ-212	423	10	2.4	Eljen	plastic
Bismuth loaded polymer	450	12	15	10.1209/0295-5075/97/22002	Plastic
bismuth+iridium loaded polymer	500	30	1200	10.1209/0295-5075/97/22002	Plastic
YAG:Ce	549	30	70	Crytur	inorganic
LuAG:Ce	535	25	70	Crytur	inorganic
YAP: Ce	370	25	25	Crytur	inorganic
CRY18	425	30	45	Crytur	inorganic
LuAG:Pr	310	18	20	Crytur	inorganic
LuAP:Ce	365	11	18	Crytur	inorganic
BGO	480	10	300	Crytur	inorganic
PWO	420	0.025	5	Crytur	inorganic
CRY19	420	24	41	Crytur	inorganic

Supplementary Table 3. Multiexponential lifetime and amplitude components of the radioluminescence decays. Three exponentials are used to fit the data with reduced chi square > 0.99.

	4.5 nm	6.0 nm	8.2 nm
A_1	0.66	0.74	0.80
t_1 (ns)	1.27	1.33	1.64
A_2	0.36	0.32	0.34
t_2 (ns)	5.53	5.05	6.95
A_3	0.19	0.11	0.11
t_3 (ns)	24.54	21.20	42.77

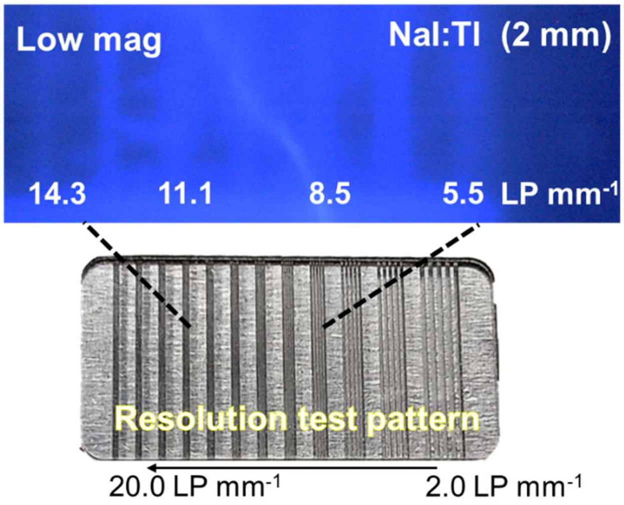

Supplementary Figure 11: **Imaging with a conventional thick scintillator.** NaI:Tl scintillator with 2 mm thickness. QS film scintillator could resolve the test patterns while NaI:Tl could not due to thickness-induced broadening.

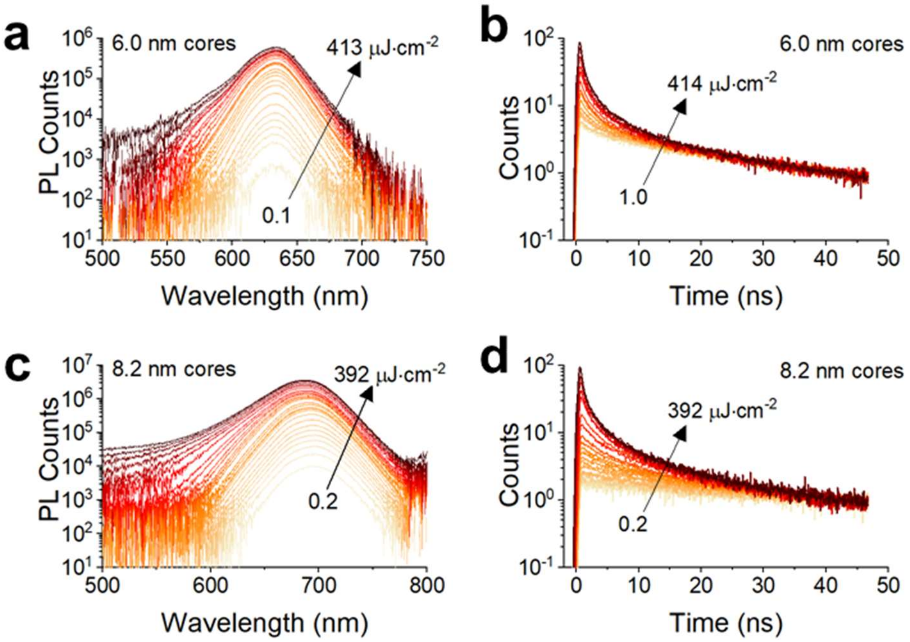

Supplementary Figure 12: **Fluence dependence photoluminescence properties.** (a) Spectra of 6.0 nm core QSs as a function of fluence with 400 nm excitation. (b) time-resolved emission of the same. (c, d) Corresponding data for 8.2 nm core QSs.

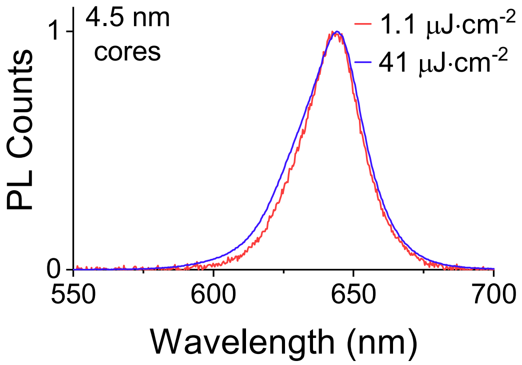

Supplementary Figure 13: **Fluence dependent photoluminescence spectra.** Normalized comparison of PL under low and modest fluence to show blue-shifted biexciton emission.

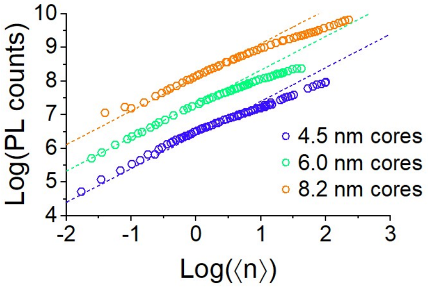

Supplementary Figure 14: **Exciton density vs. Emission intensity.** Log-log plot of the Integrated PL intensity of QS samples as a function of the average number of electron-hole pairs created under 400 nm excitation.

Supplementary Note 2: Variable power scaling model to predict radiative lifetimes of multiexcitonic states in quantum shells

The time-dependent emission intensity from an excited quantum shell is determined as a time integral across the RL contributions from all multi-exciton states, m, where $1 < m < 160$:

$$I(t) = \int_0^t dt' \sum_{m=1}^{160} \left(k_{m,\mathrm{r}} \times P(m, t') \right) \qquad (3)$$

Where $k_{m,\mathrm{r}}$ is the radiative decay rate of the m-exciton state, and $P(m, t)$ is the fraction of quantum shells with m excitons at time t.

In order to determine $P(m, t)$, we start by assuming the Poisson distribution of the initial exciton number distribution: $P(m, t = 0) = \langle N_{\mathrm{eh}} \rangle^m \times \frac{e^{-m}}{m!}$. Considering that Auger decay of an m-exciton state results in a state with $(m - 1)$ excitons, the temporal evolution of the m-exciton population in a QS, $P(m, t)$, is then determined by solving coupled rate equations:

$$\frac{dP(m,t)}{dt} = k_{m+1} P(m + 1, t) - k_m P(m, t) \qquad (4)$$

where, $k_m = k_{m,\mathrm{r}} + k_{m,\mathrm{nr}}$, represents the total (radiative + nonradiative) decay rate of an m-exciton state.

To determine multi-exciton decay rates: $k_{m,\mathrm{r}}$ and $k_{m,\mathrm{nr}}$, we use a scaling approach that was developed specifically for large-core quantum shells as detailed in *Ref.* [4]. According to aforementioned approach, the multi-exciton decay rates can be parametrized as a function of a single fitting parameter, *f*:

$$k_{m,\mathrm{r}} = m^{f-1} k_{1,\mathrm{r}} \tag{5}$$

$$k_{m,\mathrm{nr}} = m^f k_{2,\mathrm{nr}}/2^f = \frac{m^f}{2} \times \frac{1-g_{\mathrm{XX}}QY_{\mathrm{X}}}{g_{\mathrm{XX}}QY_{\mathrm{X}}} \times k_{1,\mathrm{r}} \tag{6}$$

where $g_{\mathrm{XX}} = QY_{\mathrm{XX}}/QY_{\mathrm{X}}$ is a biexciton quantum yield, which was determined to be 0.62 for 4.5-nm core QS and 0.81 for the 6.0-nm QSs (*Ref.* [5]). It was shown that f = 2.6 - 2.8 if the CdS core size is around 7-9 nm (*Ref.* [4]).

Supplementary Table 4. Parameters used for calculating decay rates in Eqs. 4 and 5

Dot		QYx	g_{XX}	tau_1(ns)	f
8.2-nm-core		0.6	0.82	110	2.7

Supplementary Table 5. Biexciton Auger lifetimes extracted from PL decay using variable-power scaling model.

Dot		QYx	g_{XX}	tau_1(ns)	f		tau_{Auger}(ns)
4.5-nm-core		0.5	0.62	49	2.7		6.78
8.2-nm-core		0.6	0.82	110	2.7		32.79

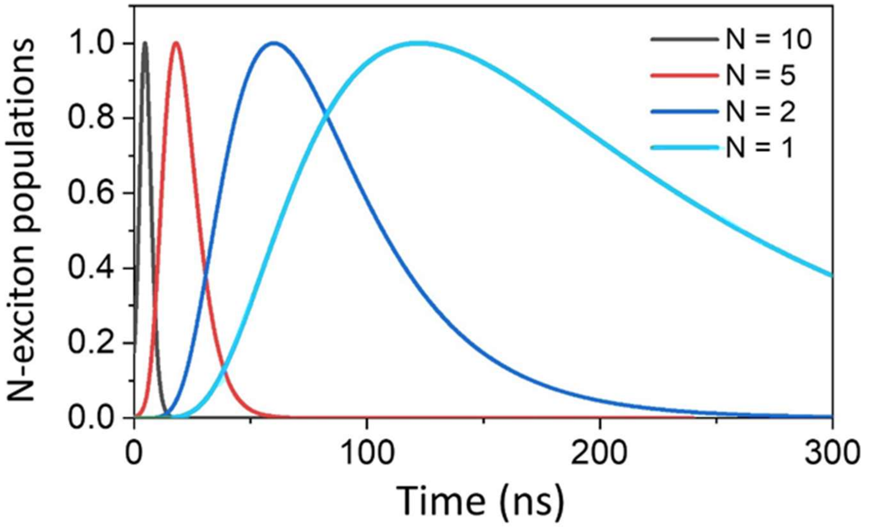

Supplementary Figure 15: **Temporal evolution of multiexciton population.** Power scaling model show the dynamic changes in exciton densities, N corresponding to a fixed number of excitons per particle, $\langle n \rangle$.

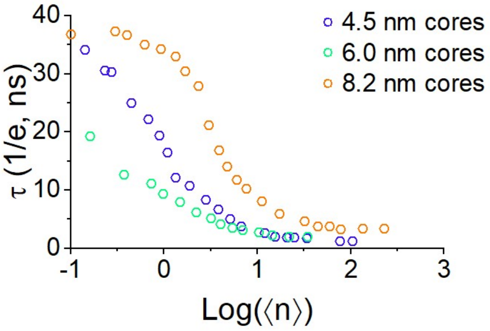

Supplementary Figure 16: **Decay lifetime vs. exciton density.** Empirical decay times (1/e time) of the time-resolved photoluminescence of the samples as a function of fluence. We find $\langle n \rangle$ that matches the measured τ_{RL}. For 4.5 nm core sample, $\langle n \rangle$ = 10. For 6.0 nm core sample, $\langle n \rangle$ = 14. For 8.2 nm sample, error bar is larger using this method, thus we refer to our multiexciton model in Figure 4e which predicts $\langle n \rangle$ = 18.

Supplementary Note 3: Estimation of the spatial spread of the absorbed X-ray photon energy across multiple quantum shells.

To obtain the spread of secondary electron deposition, we use the following experimental information and relevant deductions;

Average number of excitons $\langle N \rangle$ generated per QS for the RL state (from Figure 4e)

Emission quantum yield ($QY_{\langle N \rangle}$) of the $\langle N \rangle$ exciton state (from Figure 4b)

Total LY of the QS film, estimated by two independent methods (from Figure 1)

Based on this information, we calculate how many QSs (n_{QS}) are involved in the RL.

Emitted photons based on LY; $n_{\mathrm{emit\_photons}} = LY \times E_{\mathrm{x-ray}} = 69\,\frac{photons}{keV} \times 11.5\ keV =$ 793 photons

Emitted photons based on $\langle n \rangle$ and $QY_{\langle n \rangle}$; $n_{\mathrm{emit\_photons}} = n_{\mathrm{QS}} \times \langle n \rangle \times QY_{\langle n \rangle} = n_{\mathrm{QS}} \times 18 \times 0.3$

We estimate n_{QS} to be 147, representing the number of QSs excited by subsequent secondary electron processes. Considering that the total size of a QS is ~12 nm, we estimate the diameter of the secondary electron spread to be 126 nm for a close packed QS film. Supplementary Figure 17 shows a sketch of the secondary electron deposition across a close-packed QS film.

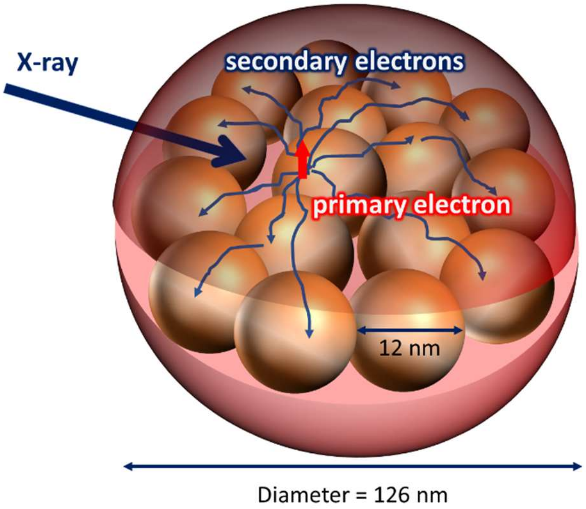

Supplementary Figure 17: **Secondary electron deposition.** The extent of secondary electrons reaching ca. 135 nm in diameter for a close-packed QS film.

Supplementary Note 4: Cathodoluminescence properties of quantum shells

Colloidal quantum shells also show strong, ultrafast cathodoluminescence (CL) under electron irradiation (see Methods). The measured CL spectra in Supplementary Figures 18 and 19. Supplementary Figure 18 shows QS CL in comparison to that of a gold film, a CL standard with predictable, coherent d-band emission.[6] The QS CL is substantially stronger than gold film. Supplementary Figure 19b shows the CL spectrum of 8.2 nm QS with respect to gold. The PL vs. CL peak positions are the same (Supplementary Figure 19a). Importantly, the CL lifetimes are quite fast, ranging around 100 – 280 ps (Supplementary Figure 18b). These CL lifetimes are much faster than the RL lifetimes. It is unclear if this may be related to additional charging of the QSs under direct electron excitation. Understanding this would require future work.

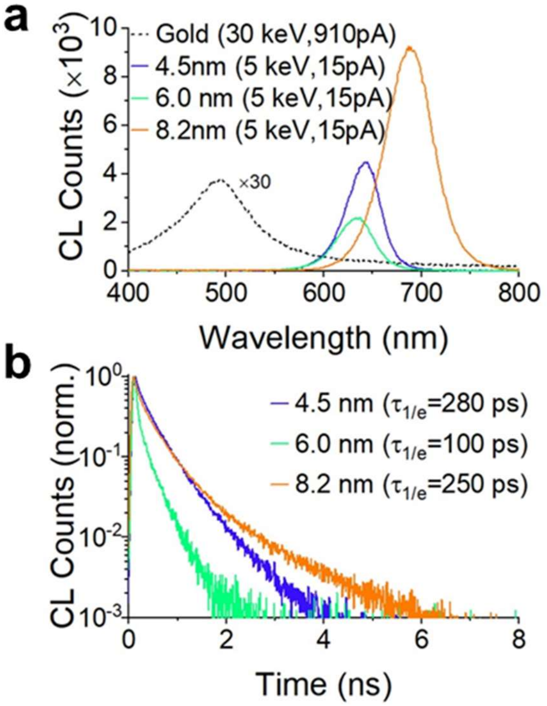

Supplementary Figure 18: **Cathodoluminescence properties.** (a) Cathodoluminescence of QS in comparison to a gold film reference sample. (b) Time-resolved cathodoluminescence of the same QS samples.

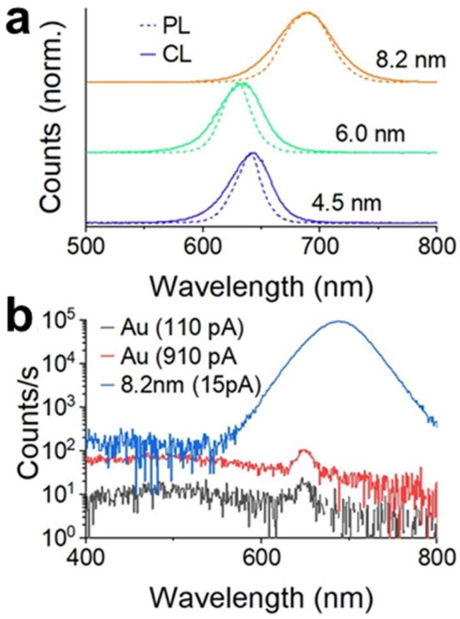

Supplementary Figure 19: **Cathodoluminescence vs. Photoluminescence.** (a) Comparison of photoluminescence (PL) and cathodoluminescence (CL) form QSs. (b) Comparison of 8.2 nm core QS CL versus CL from gold films, with all measurements at 5 keV accelerating voltage.

Supplementary References:

1. Moszynski, M., Kapusta, M., Mayhugh, M., Wolski, D. & Flyckt, S. O. Absolute light output of scintillators. *IEEE Trans Nucl Sci* **44**, 1052–1061 (1997).
2. Mazziotta, M. N. Electron–hole pair creation energy and Fano factor temperature dependence in silicon. *Nucl Instrum Methods Phys Res A* **584**, 436–439 (2008).
3. Gather, M. C. & Reineke, S. Recent advances in light outcoupling from white organic light-emitting diodes. *J Photonics Energy* **5**, 057607 (2015).
4. Harankahage, D. *et al.* Quantum Shell in a Shell: Engineering Colloidal Nanocrystals for a High-Intensity Excitation Regime. *J Am Chem Soc* **145**, 13326–13334 (2023).
5. Cassidy, J. *et al.* Quantum Shells Boost the Optical Gain of Lasing Media. *ACS Nano* **16**, 3017–3026 (2022).
6. Brenny, B. J. M., Coenen, T. & Polman, A. Quantifying coherent and incoherent cathodoluminescence in semiconductors and metals. *Journal of Applied Physics* **115**, 244307 (2014).
